# An Unusual Appearing Skin Lesion from *Penicillium marneffei* Infection in an AIDS Patient in Central China

**DOI:** 10.4269/ajtmh.14-0697

**Published:** 2015-07-08

**Authors:** Yanni Xiang, Wei Guo, Ke Liang

**Affiliations:** Department of Infectious Diseases, Zhongnan Hospital of Wuhan University, Wuhan, China; Pathology Department, Wuhan University School of Basic Medical Sciences, Wuhan, China

**Figure 1.**
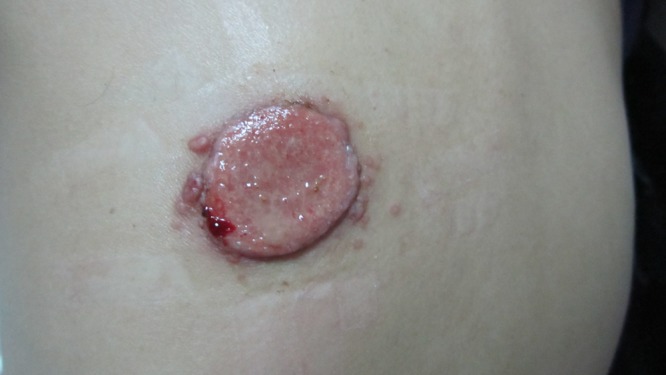
The skin lesion on the patient's left back.

**Figure 2.**
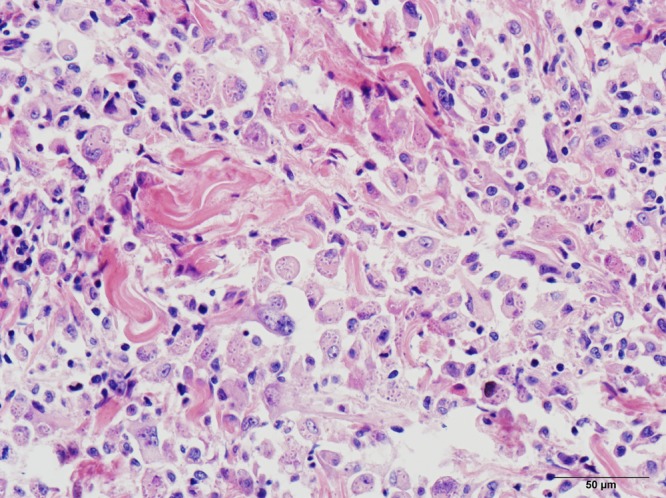
Skin biopsy showing numerous intracellular round to oval yeast-form cells (hematoxylin and eosin, ×400).

A 46-year-old woman presented with a slowly enlarging skin lesion on the back, jugular lymphadenopathy for 1 month, but no fever. She was infected with the human immunodeficiency virus (HIV); CD4+ T lymphocyte count was 22 cells/mm^3^. She had always lived in Hubei province (central China) and denied a history of traveling to southern China. The skin lesion was 3 × 3 cm flesh-colored, well-demarcated, and ulcerated (Figure 1). Lymph node biopsy showed granulomatous inflammation. Bone marrow smear did not show specific pathological changes. Blood and bone marrow cultures were negative, but skin biopsy stained with hematoxylin and eosin (Figure 2) and periodic acid–Schiff revealed yeast-like microorganisms. Mycological culture of skin biopsy showed *Penicillium marneffei*. Intravenous amphotericin B led to full recovery. *P. marneffei* causes penicilliosis, an opportunistic infection geographically restricted to Southeast Asia and southern China. In a study of penicilliosis patients in mainland China from 1984 to 2009, nearly 100% of the patients had a history of traveling or residing in the south.^1^ The classic skin lesion—papules with central necrotic umbilication—had always served as an important clue for the diagnosis of *P. marneffei* infection.^2^ In this case, the residence of the patient outside southern China and the unusual appearance of the skin lesion made diagnosis difficult. This patient's history suggests that endemic areas of *P. marneffei* may extend beyond Southeast Asia and southern China. The negative blood and bone marrow cultures and non-specific lymph node biopsy indicate that this patient did not have a disseminated infection typical of acquired immune deficiency syndrome (AIDS) patients with penicilliosis. Biopsy and fungal cultures of skin lesions are useful for diagnosing penicilliosis.
